# Prevalence of hepatitis B and delta according to HIV-type: a multi-country cross-sectional survey in West Africa

**DOI:** 10.1186/s12879-017-2568-5

**Published:** 2017-07-04

**Authors:** Patrick A. Coffie, Boris K. Tchounga, Guillaume Bado, Mathieu Kabran, Daouda K. Minta, Gilles Wandeler, Geoffrey S. Gottlieb, François Dabis, Serge P. Eholie, Didier K. Ekouevi

**Affiliations:** 1 Département de Dermatologie et d’Infectiologie, UFR des Sciences Médicales, Université Félix Houphouët Boigny, BP V3 Abidjan, CHU de Treichville, Abidjan, Côte d’Ivoire; 2 Service des Maladies Infectieuses et Tropicales, Centre Hospitalier Universitaire de Treichville, Abidjan, Côte d’Ivoire; 3 Programme PACCI, site de recherche ANRS, Abidjan, Côte d’Ivoire; 4 ISPED, Université de Bordeaux & Centre INSERM U1219 - Bordeaux Population Health, Bordeaux, France; 5 Hôpital de Jour, Service des Maladies Infectieuses et Tropicales, CHU Souro Sanou, Bobo Dioulasso, Burkina Faso; 6 Département d’Hématologie, d’immunologie et de biologie cellulaire, UFR des Sciences Pharmaceutiques, Université Félix Houphouët Boigny, Abidjan, Côte d’Ivoire; 7 Centre de Prise en Charge des Personnes vivant avec le VIH, Service de Maladies Infectieuses, Hôpital du Point G, Bamako, Mali; 8 Department of Infectious Diseases, Bern University Hospital, University of Bern, Bern, Switzerland; 9 Institute of Social and Preventive Medicine, University of Bern, Bern, Switzerland; 10 Departments of Medicine & Global Health, University of Washington, Seattle, USA; 11 Université de Lomé, Département des Sciences Fondamentales et Santé Publique, Lomé, Togo

**Keywords:** HIV-2, HBV, HBV and HDV coinfection, West Africa

## Abstract

**Background:**

In West Africa where HIV-1 and HIV-2 co-circulate, the co-infection with hepatitis B virus (HBV) and hepatitis Delta virus (HDV) is not well described. This study aimed at estimating the prevalence of HBV and HBV/HDV co-infection according to HIV types and risk factors for HBV infection among West African HIV-infected patients.

**Method:**

A cross-sectional survey was conducted within the IeDEA West Africa cohort from March to December 2012 in Côte d’Ivoire (three sites), Burkina Faso and Mali (one site each). All HIV-infected adult patients on antiretroviral therapy (ART) or not who attended one of the participating HIV clinics during the study period and agreed to participate were included. Blood samples were collected and re-tested for HIV type discrimination, HBV and HDV serology as well as HBV viral load. Logistic regression was used to identify risk factors for HBV infection.

**Results:**

A total of 791 patients were included: 192 HIV-1, 447 HIV-2 and 152 HIV-1&2 dually reactive. At time of sampling, 555 (70.2%) were on ART and median CD4+ cell count was 472/mm^3^ (inter-quartile range [IQR]: IQR: 294–644). Sixty-seven (8.5%, 95% CI 6.6–10.6) patients were HBsAg positive without any difference according to HIV type (7.9% in HIV-1, 7.2% in HIV-1&2 dually reactive and 9.4% in HIV-2; *p* = 0.61). In multivariate logistic analysis, age ≤ 30 years old (adjusted odds ratio [aOR] 5.00, 95% CI 1.96–12.76), age between 31 and 49 years old (aOR 1.78, 95% CI 1.00–2.21) and male gender (aOR 2.15, 95% CI 1.25–3.69) were associated with HBsAg positivity. HBV DNA testing was performed in 36 patients with blood sample available (25 on ART) and 8 (22.2%) had detectable HBV DNA. Among the HBsAg-positive individuals, 14.9% (95% CI 7.4–25.7) were also positive for anti-HDV antibody without any difference according to HIV type (28.6% in HIV-1, 14.3% in HIV-2 and 0.0% in HIV-1&2 dually reactive; *p* = 0.15).

**Conclusion:**

HBV and HBV/HDV co-infection are common in West Africa, irrespective of HIV type. Therefore, screening for both viruses should be systematically performed to allow a better management of HIV-infected patients. Follow-up studies are necessary to determine the impact of these two viruses on HIV infection.

## Background

In West Africa, approximately 8% of HIV-infected individuals have a chronic hepatitis B virus (HBV) infection [1]. In this part of the world, HBV infection is one of the main causes of end-stage liver disease, cirrhosis and hepatocellular carcinoma (HCC) [2].

West Africa is also characterized by the circulation of both HIV type 1 and 2, which can to lead to co-infections with HIV-1 and HIV-2 (HIV-1&2) [3–5]. Compared to HIV-1 infection, HIV-2 infection is characterized by a longer asymptomatic phase and a slower disease progression [6]. The management of HIV-2 infection is more challenging than the one of HIV-1, due to the intrinsic resistance of HIV-2 to non-nucleoside reverse transcriptase inhibitors (NNRTIs) and reduced sensitivity to several protease inhibitors (PIs) [7, 8].

Due to shared routes of transmission, HIV and HBV co-infection is common and 5–20% of HIV-infected people have also chronic HBV infection worldwide [9]. Compared to HBV or HIV-mono-infected individuals, HIV/HBV-co-infected patients have a higher risk of impaired immunological recovery and hepatotoxicity during antiretroviral treatment (ART) [10–12] and a faster rate of progression to cirrhosis and HCC [9]. Thus, knowledge of HBV status among HIV-infected patients is important for clinical monitoring and selection of ART, as tenofovir disoproxil fumarate (TDF) and lamivudine (3TC) or emtricitabine (FTC) should be part of the treatment [13].

In West Africa, little is known in about the epidemiology of hepatitis D virus (HDV) infection, a defective RNA virus that requires the presence of HBV to infect the hepatocytes [14]. Its impact on HIV/HBV co-infected patients is not documented since HDV is not routinely diagnosed. It has been estimated that approximately 5% of HBV carriers are co-infected with HDV worldwide [15, 16] and between 3% and 25% in West Africa [17–20]. HDV co-infection increases the risk for hepatitis flares and chronic hepatic complications [15, 21] and patients with HBV/HDV co-infection have a significantly increased risk for HCC compared with patients with HBV mono-infection and the general population [22]. Therefore, HDV screening is very important for the monitoring of HIV/HBV co-infected patients.

To our knowledge, only one small-scale study from Guinea Bissau has estimated the prevalence of HDV/HBV co-infection according to HIV types and did not find any difference between HIV-1 and HIV-2 [19]. However, this needs to be confirmed with a large and multicenter study*.* The objectives of this study were to estimate the prevalence of HBV and HBV/HDV co-infection according to HIV types among a large series of HIV-infected patients in the WADA (West Africa Database on Antiretroviral Therapy) cohort in three West African countries and, to identify risk factors for HBV seropositivity.

## Methods

### Study design and settings

A cross-sectional survey was conducted from March to December 2012 in three countries (Burkina Faso, Côte d’Ivoire and Mali) within the WADA cohort. This cohort is embedded in the International epidemiological Database to Evaluate AIDS (IeDEA) West Africa Collaboration, which is part of the global IeDEA network [23].

### Study population

All patients aged 18 years and above, registered in the WADA cohort as HIV-2 or dually reactive, who attended one of the participating clinics during the study period and who agreed to participate were included in this survey regardless of ART initiation according to WHO 2010 guidelines [24].

### Data collection

A standardized survey form was used to collect data on patients’ demographics, clinical and biological characteristics. Two EDTA tubes of blood were collected from each patient and sent to the referral laboratory of the study (CeDReS, Treichville Hospital in Abidjan, Côte d’Ivoire) to perform HIV type discrimination and hepatitis analyses.

### HIV retesting

All patients identified as HIV-2 or dually reactive on clinical site according to the national algorithms were screened de novo with two immuno-enzymatic tests: Immunocomb II HIV 1 & 2 BISPOT (Orgenics Ltd. Yavne, − Alere), a World Health Organization (WHO)-endorsed indirect, immuno-enzymatic test (sensitivity 100%; specificity 99%) [25] and an in-house ELISA test, developed by the French National Aids and Viral Hepatitis Research Agency (ANRS) [26]. The results of this rescreening were previously reported [27]. The aim of this retesting was to perform an accurate HIV type discrimination, since HIV type misclassification has previously been reported in many West African cohorts, especially for HIV-1&2 dually reactive patients [27, 28].

### HBV and HDV measurements

Qualitative HBsAg was detected using Monolisa® HBsAg ULTRA (Bio-Rad, Evolis Tween Plus, Marnes- la- Coquette, France), a one-step sandwich enzyme immunoassay. Samples reactive for HBsAg were subsequently tested for HBV DNA and HDV serology. All tests were performed according to manufacturer’s instructions. The quantitative measurement of HBV DNA in plasma was done with the COBAS® AmpliPrep/COBAS® TaqMan® HBV Test (Roche Molecular Systems, Inc. Roche Diagnostics GmbH). The limit of detection of this assay was 20 IU/ml. Testing for anti-HDV antibody was performed using ETI-AB-DELTAK-2, an enzyme immune-assay for the qualitative determination of total antibodies to hepatitis delta antigen (anti-HD) (DiaSorin Limited, United Kingdom).

### Statistical analyses

Continuous variables were described with median and interquartile range (IQR) and categorical variables as percentages. The prevalence of HBV and HDV infections was expressed with a 95% confidence interval (95% CI). Group’s comparisons were performed using Student’s *t* test or non-parametric Wilcoxon rank-sum test (non-normal distribution) for continuous variables and using Chi-2 test or Fisher’s exact test for categorical variables. Univariable and multivariable logistic regression analyses were performed with a stepwise-descending selection procedure to identify risk factors of HBsAg positivity. The selection of covariates for multivariable analysis was based on the univariable analyses with factors associated with HBsAg positivity (*p* < 0.25). Adjusted Odds Ratios (aORs) were reported with 95% CI. We deemed a *p*-value <0.05 as statically significant for all analyses. Data analyses were performed using Stata software (Stata™ 11.0 College Station, Texas, USA).

### Ethics

This survey was designed and performed in accordance with the Declaration of Helsinki and was approved by the national ethics committee of each participating country: the “Comité d’Ethique pour la Recherche en Santé” (Ministry of Health and Ministry of Scientific Research and Innovation) from Burkina-Faso, the “Comité National pour l’Ethique et la Recherche en Santé” (Ministry of Health and the Fight against HIV/AIDS) from Côte d’Ivoire and the “Comité National d’Ethique pour la Santé et les Sciences de la vie (Ministry of Health)” from Mali. All patients were informed and had to give their written consent before being included.

## Results

### Study population

From March to December 2012, 791 HIV-infected patients were included in this study: 232 (29.3%) from Burkina Faso, 535 (67.7%) from Côte d’Ivoire and 24 (3.0%) from Mali. After the retesting for HIV type discrimination, 192 (24.3%) patients were classified as HIV-1, 447 (56.5%) HIV-2 and 152 (19.2%) as HIV-1&2 dually reactive. At time of sample collection, the overall median age was 47 years [IQR): 40–53], 472 (59.7%) were women; median CD4+ cell count was 472 cells/mm^3^ [IQR: 294–644]; 555 (70.2%) were on ART, of whom 522 (94.0%) had initiated a regimen composed of two nucleoside reverse transcriptase inhibitors (NRTIs) and one PI. Approximately three-quarters of patients (73.2%) were taking an ART regimen containing 3TC (or FTC) without TDF and 21.8% an ART regimen containing TDF and 3TC (or FTC). Median duration on ART was 3.6 years [IQR: 1.9–6.2]. Table 1 summarizes characteristics of patients at the time of blood collection.

**Table 1:** Characteristics of patients at time of blood collection by HIV status – Burkina Faso, Côte d’Ivoire and Mali – IeDEA West Africa cohort (2012)

Variables	Total	HIV-1	HIV-2	HIV-1&2	*P*-value
	*N* = 791	*N* = 192	*N* = 447	*N* = 152	
Age (years)					
Median [IQR]	47 [40–53]	45 [37–51]	49 [42–54]	47 [42–52]	0.0004
≤ 30	40 (5.1)	14 (7.3)	25 (5.6)	1 (0.7)	<0.001
31–49	438 (55.4)	112 (58.3)	224 (50.1)	102 (67.1)	
≥ 50	313 (39.5)	66 (34.4)	198 (44.3)	49 (32.2)	
Gender					
Male	319 (40.3)	76 (39.9)	192 (43.0)	51 (33.6)	0.12
Female	472 (59.7)	116 (60.4)	255 (57.1)	101 (66.5)	
Country					
Burkina Faso	232 (29.3)	70 (36.4)	81 (18.1)	81 (53.3)	<0.001
Côte d’Ivoire	535 (67.7)	116 (60.4)	350 (78.3)	69 (45.4)	
Mali	24 (3.0)	6 (3.1)	16 (3.4)	2 (1.3)	
CD4 count (cells/mm^3^)					
Median [IQR]	472 [294–644]	405 [276–599]	488 [311–675]	448 [295–615]	0.0001
≤ 200	115 (14.5)	32 (16.7)	64 (14.3)	19 (12.5)	0.54
> 200	676 (85.5)	160 (83.3)	383 (85.7)	133 (87.5)	
Antiretroviral treatment					
No	236 (29.8)	20 (10.4)	191 (42.7)	25 (16.5)	<0.0001
Yes	555 (70.2)	172 (89.6)	256 (57.3)	127 (83.6)	
Antiretroviral regimen^a^					
Triple NRTI	26 (4.7)	8 (4.7)	13 (5.1)	5 (3.9)	0.95
NRTI + NNRTI	7 (1.3)	3 (1.7)	3 (1.2)	1 (0.8)	
NRTI + PI	522 (94.0)	161 (93.6)	240 (93.7)	121 (95.3)	
Antiretroviral drugs^a^					0.34
3TC alone	406 (73.2)	131 (76.1)	177 (69.2)	98 (77.2)	
TDF + 3TC (FTC)	121 (21.8)	34 (19.8)	62 (24.2)	25 (19.7)	
Others	28 (5.0)	7 (4.1)	17 (6.6)	4 (3.1)	

*IQR* Interquartile range, *NRTI* nucleoside reverse transcriptase inhibitor, *NNRTI* Non-nucleoside reverse transcriptase inhibitor, *PI* Protease inhibitor

^a^Among patients on ART only

### HBV serology

Sixty-seven patients were tested positive for HBsAg, giving an overall prevalence of 8.5% (95% CI 6.6–10.6). HBsAg prevalence did not significantly vary according to country (9.1% in Burkina Faso, 8.3% in Mali and 8.2% in Côte d’Ivoire, *p* = 0.91) or HIV type (7.9% in HIV-1, 7.2% in HIV-1&2 dually reactive and 9.4% in HIV-2; *p* = 0.61), but varied according to gender (11.3% in males vs. 6.6% in females; *p* = 0.02) (Table 2).

**Table 2:** Risk factors for HBsAg seropositivity

			Univariate analysis			Multivariate analysis		
Factor	No. of patients with data	HBsAg positivity	OR	95% CI	*P*	aOR	95% CI	*P*
Age (years)								
≤ 30	40	8 (20.0)	3.48	1.42–8.48	0.006	5.00	1.96–12.76	0.001
31–49	438	38 (8.7)	1.32	0.76–2.30	0.33	1.78	1.00–2.21	0.05
≥ 50	313	21 (6.7)	REF	-	-	REF	-	-
Gender								
Male	319	36 (11.3)	1.81	1.09–2.99	0.02	2.15	1.25–3.69	0.005
Female	472	31 (6.6)	REF	-	-	REF	-	-
HIV-type								
HIV-2	447	42 (9.4)	1.59	0.77–3.27	0.21	1.66	0.85–3.26	0.14
HIV-1/2	152	11 (7.2)	1.10	0.44–2.72	0.84	1.11	0.48–2.56	0.81
HIV-1	192	14 (7.3)	REF	-	-	REF	-	-
Country								
Burkina Faso	232	21 (9.1)	1.11	0.64–1.91	0.71	1.30	0.72–2.36	0.38
Mali	24	24 (8.3)	1.01	0.23–4.46	0.98	1.01	0.20–4.34	0.93
Côte d’Ivoire	535	44 (8.2)	REF	-	-	REF	-	-
CD4 count (cells/mm^3^)								
≤ 200	115	13 (11.3)	1.47	0.77–2.79	0.24	1.43	0.72–2.84	0.31
> 200	676	54 (8.0)	REF	-	-	REF	-	-
Antiretroviral treatment								
Yes	555	51 (9.2)	1.39	0.78–2.49	0.24	1.56	0.83–2.95	0.17
No	236	16 (6.8)	REF	-	-	REF	-	-

*OR* Odds ratio, *CI* Confidence Interval, *aOR* Adjusted Odds ratio

Among the HBsAg-positive individuals, 51 (76.1%) were on ART: 48 (94.1%) on a PI-based regimen, two (3.9%) on a NNRTI-based regimen and one (2.0%) on a triple NRTI-based regimen. Thirty-one patients (60.8%) on ART were receiving 3TC (or FTC) without TDF and 17 (33.3%) patients were on TDF + 3TC.

In multivariate analysis adjusting on HIV type, country, CD4 cell count and ART (Table 2), factors significantly associated with HBsAg positivity were male gender (aOR 2.15, 95% CI 1.25–3.69), age ≤ 30 years old (aOR 5.00, 95% CI 1.96–12.76) and 31–49 years old (aOR 1.78, 95% CI 1.00–2.21) (Table 2).

### HBV viral characteristics

Among 67 HBsAg-positive individuals, 36 (53.7%) had a blood sample available for HBV DNA measurement and among them, 25 (69.4%) were on ART including 16 on 3TC and seven on TDF plus 3TC. There were no significant differences in socio demographic characteristics between patients with and without HBV DNA measurement, except for HIV type (85.7% of HBV DNA measurement in HIV-1 infected patients, 40.5% in HIV-2 and 63.6% in HIV-1&2, *p* = 0.01). Overall, HBV DNA was detected in eight of 36 patients (22.2%) and the median HBV DNA was 42,050 IU/ml [IQR: 51–79,100,000]. The proportion of patients with undetectable HBV DNA was not significantly different according to ART regimen (100.0% with TDF + 3TC (or FTC), 87.5% with 3TC and 50.0% with ART without 3TC or TDF; *p* = 0.25). The detection rate was not significantly different according to HIV type (25.0% in HIV-1, 23.5% in HIV-1&2 dually reactive and 14.3% in HIV-2; *p* = 1.00), while it was significantly higher in patients not on ART compared to those on ART (45.5% vs 12.0%; *p* = 0.04).

### HDV co-infection

Ten (14.9%, 95% CI 7.4–25.7) of the 67 HBsAg-positive individuals were tested positive for anti-HDV antibody. Six of them were on ART, including three on 3TC and one on TDF + 3TC (FTC). There was no statistical difference in anti-HDV antibody prevalence according to type of HIV, although the proportion tended to be high among HIV-1 infected patients (28.6% in HIV-1, 14.3% in HIV-2 and 0.0% in HIV-1&2 dually reactive; *p* = 0.15). There was also no statistical difference in this prevalence according to all the other characteristics displayed in Table 1. The prevalence of anti-HDV antibody positivity did not differ according to HBV DNA detectability (21.4% in patients with a suppressed HBV DNA vs 25.0% in those with a detectable HBV DNA; *p* = 1.00).

## Discussion

In this cross-sectional survey, the prevalence of HBV infection and HBV/HDV co-infection were relatively high, with no statistical difference by country and HIV types. To our knowledge, this is the largest report up to now on HBV/HDV co-infection among HIV-infected individuals in West Africa and the first in the three participating countries (Burkina Faso, Côte d’Ivoire and Mali). There are indeed few published studies in this part of the world [17–19], and none of them, except in Guinea Bissau, has estimated the prevalence by HIV type [19].

Our study has some limitations. First, about 19% of the patients were considered as HIV-1 & HIV-2 dually reactive after the retesting. We were not able to differenciate between HIV-1 or type 2 since we did not perform PCR DNA for HIV-1 or HIV-2. Second, we did not document occult HBV infection since HBV DNA was only tested in HBsAg-positive patients. This has probably underestimated the prevalence of HBV infection. Indeed, in one study conducted in Côte d’Ivoire, 10% of HBsAg-negative patients had detectable HBV DNA [29]. Third, the lack of molecular data concerning the HDV as HDV RNA testing has become a more compelling tool for recognizing active replication. This may have overestimated the prevalence of HDV co-infection. Fourth, due to low amounts of plasma available, HBV DNA was not performed in all HBsAg-positive patients, and resistance and genotypic tests were not performed. In West Africa region, Genotype E is the predominant genotype [30]. Finally, our study population was selected based on the local identification of HIV-2 and dually reactive patients. The HIV-1 subgroup may thus not be fully representative of all HIV-1 patients in care in the same clinics. Despite these limitations, this study has also two major strengths. First it took place in three different West African countries and second it included a large sample of HIV-infected patients, giving therefore for the first time a global view of the distribution of HBV and HBV/HDV co-infection by HIV type in West Africa.

In our study, the prevalence of HBV was 8.5% (95% CI 6.6–10.6) among HIV-infected patients. This result is consistent with previous studies conducted in West Africa that have estimated the prevalence of HBV between 7.9% and 16.8% [17, 19, 31–33]. The variation of this prevalence according to studies is possibly due to the HBV vaccination coverage and the specific distribution of some risk factors for HBV infection. Indeed we found that male patients and those <50 years old were more likely to be infected with HBV, which is consistent with previous reports [20, 34–36]. The high prevalence of HBV in these two groups could be explained by a greater exposure to HBV through sexual behavior and body mutilations [20, 34, 35], but also by the early death of older patients infected with HBV during childhood. Indeed, in sub-Saharan Africa, 25% of adults who have been infected during childhood die from cirrhosis or liver cancer [37]. Other risk factors for HBV infection, such as WHO stage 3 or 4 and CD4 cell count <200 cells/mm^3^, were also found in other studies [36].

Only one-third of the patients co-infected with HIV and HBV received a standard-of-care ART regimen including TDF and 3TC (or FTC), two drugs active against both HIV and HBV. The remaining two-thirds were only exposed to lamivudine for about four years, a situation which is known to lead to the development of lamivudine-resistant HBV, from 43% in year 1 to >80% in year 4 [38–40]. Those patients with long-standing lamivudine-resistant mutations may experience worsening liver disease. Since 2013, WHO recommends TDF and 3TC (or FTC) as the preferred NRTI option in first-line ART regimen in adolescents and adults infected with HIV [13, 41], and this recommendation is gradually endorsed by countries. This should allow an optimal management of HIV and HBV co-infection, including occult hepatitis. Indeed, according to a recent meta-analysis, up to 90% of patients receiving such a treatment had a suppressed HBV DNA after three years [42] and resistance to TDF has not yet been described in vivo [43, 44]. However, even with WHO guidelines for ART use in HIV*-*infected individuals, including two molecules active on both HIV and HBV, the benefits of HBV screening is still important, including referral for HBV vaccination in susceptible individuals and a better management of those already infected with HBV disease.

Regarding HDV infection, previous studies in sub-Saharan Africa have reported a prevalence of HBV/HDV co-infection ranging between 0.0% and 44.4% [17, 18, 20, 45, 46]. More recently, one study conducted in Guinea Bissau found a high prevalence of HDV/HBV co-infection (25.0%), with no statistical difference according to HIV type [19]. In our study, the HBV/HDV prevalence was 14.9%, not different by HIV type. This large variation of HDV prevalence could be explained by the different settings and populations, but also by the tests used for HDV diagnosis. Indeed, in one cohort of over 200 HIV/HBV co-infected individuals in rural Tanzania, no confirmed case of active HDV infection (second serology and nucleic acid amplification) was found among the 11 patients who had a positive anti-HDV antibody screening test [46]. Thus, all anti-HDV antibody positive samples should be confirmed with an additional antibody test or an HDV RNA test if possible. The management of this triple infection is complicated. Indeed, the effect of TDF alone or in association with 3TC (or FTC) on HDV replication is controversial according to studies [47, 48]. More research and tools are needed to improve our knowledge on the distribution and management of HBV/HDV co-infection among HIV-infected patients.

Finally, this large and multicenter study showed that like in Guinea Bissau, there was no statistical difference of the prevalence of HBV or HBV/HDV according to HIV types [19]. Thus, the specificity of HIV-2 infection seems to have no impact on the transmission and the rate of HBV and HBV/HDV infection.

## Conclusions

HBV and HBV/HDV co-infection seem to be common in West Africa, irrespective of HIV type and the country. Therefore, the screening of both viruses should be systematically performed in HIV-infected patients to allow a better management of HIV. Follow-up studies are needed to improve our knowledge on HDV infection and to determine the impact of these two viruses on the course of HIV infection, especially now with the universal test and treat policy.

## Abbreviations

ANRS: Agence Nationale de la Recherche sur le VIH/Sida et les Hépatites Virales
aOR: Adjusted Odd Ratio
ART: Antiretroviral treatment
CeDReS: Centre de Diagnostic et de Recherche sur le Sida et les maladies associées
CERS_BF: Comité d’Ethique pour la Recherche en Santé au Burkina Faso
CI: Confidence Interval
CNER_CI: Comité National pour l’Ethique et la Recherche en Côte d’Ivoire
DNA: Deoxyribonucleic Acid
ELISA: Enzyme Linked Immunosorbent Assay
HBV: Hepatitis B virus
HCC: Hepatocellular Carcinoma
HDV: Hepatitis Delta virus
HIV: Human Immunodeficiency Virus
HIV-1: HIV type 1
HIV-1&2: HIV type 1 &2
HIV-2: HIV type 2
IeDEA: International Epidemiological Database to Evaluate Aids
IQR: Interquartile Range
OR: Odd Ratio
PCR: Polymerase Chain Reaction
PI: Protease Inhibitors
RNA: Ribonucleic Acid
TDF: Tenofovir Disoproxil Fumarate
WADA: West Africa Database on Antiretroviral
